# GNSS Based Low-Cost Magnetometer Calibration

**DOI:** 10.3390/s22218447

**Published:** 2022-11-03

**Authors:** Ján Andel, Vojtech Šimák, Alžbeta Kanálikova, Rastislav Pirník

**Affiliations:** Department of Control and Information Systems, Faculty of Electrical Engineering and Information Technology, University of Žilina, 010 26 Žilina, Slovakia

**Keywords:** magnetometer, data acquisition unit, calibration, GNSS

## Abstract

With the development of MEMS sensors, the magnetometer has increasingly become a part of various wearable devices. The magnetometer measures the intensity of the magnetic field in all three axes, resulting in a 3D vector—direction and power. Calibration must be done before using a magnetometer, especially in wearable electronics, due to the low quality of the sensor and high proximity to other electromagnetic emission sources. Several magnetometer calibration algorithms exist in the literature, with most of them requiring multi-sided rotation. However, such calibration is highly impractical when the sensor is mounted on larger objects, e.g., vehicles, which cannot easily be rotated. Vehicles contain a large amount of ferromagnetic soft and hard material that affects the measured magnetic field. A magnetometer can be useful for an INS system in a car as long as it does not drift over time. This article describes how to calibrate a magnetometer using the GNSS motion vector. The calibration is performed using data from the initial section of the vehicle’s trajectory. The quality of the calibration is then validated using the remaining section of the trajectory, comparing the deviation between the azimuth obtained by GNSS and by the calibrated magnetometer. Based on the azimuth and speed of the vehicle, we predicted the position of the vehicle and plotted the prediction on the map. The experiment showed that such calibration is functional. The uncalibrated data were unusable due to the strong effect of ferromagnetic soft and hard materials in the vehicle.

## 1. Introduction

The application area of the magnetometer is broad. However, the Earth’s magnetic field is weak, and its measurement is often distorted due to ferromagnetic materials in the surroundings. Compasses on ships have also had to be calibrated and compensated since iron ships began to be made. Their calibration is ensured using two iron balls on the sides. The magnetic compass should be calibrated every time before use. There are a wide variety of different approaches to calibrating a magnetometer. Overall, there are two groups of methods: offline and online calibration. With offline calibration, we get calibration data that we do not change afterward; with online calibration, we continuously get current calibration data [1]. Such an approach is better because the disturbing influences around the sensor can change (e.g., electrical current flowing through the conductor around the sensor, movement of the ferromagnetic material in the surroundings). However, online calibration is not always possible. Finally, during offline calibration, it is necessary to rotate the sensor in all directions.

The most straightforward approach to obtain calibration constants is to get minima and maxima in individual axes after rotation. Due to the rotation, we get a cloud of points that look like an ellipsoid. The average value from the min and max will determine the calibration value of the offset in the given axis. However, such an approach is trivial. We only get offset values. A more common approach is the search for an optimal ellipsoid fitting the cloud of points which determines the calibration constants. Such an approach is described in several articles [2,3,4,5]. Some researchers solved the search for ellipsoids via maximum likelihood (ML) estimation [6]. Online calibration is mostly based on the Kalman filter and its modifications [7,8] or through non-linear programming [1]. For online calibration, the recursive least square (RLS) estimation and ML estimation methods can be used [9]. Such an approach is also suitable for other three-axis sensors. Several algorithms have been developed based on the discontinuity between the axes of different sensors (accelerometer and gyroscope) [10,11,12]. It is possible to calibrate the magnetometer based on the gyroscope and accelerometer. Authors in Reference [13] calibrated the magnetometer and gyroscope to each other, and the error of the magnetometer was reduced to 0.5° while the magnetometer and the gyroscope were mounted on the same platform [13]. We can also use neural networks to calibrate the magnetometer using a gyroscope, but such an approach is computationally demanding [14]. Rotating vehicles such as cars, ships, or airplanes is difficult. There are magnetometer calibration approaches that calibrate based on level rotation [15]. Such an approach has also proven to be very effective. The magnetometer in small drones can be calibrated based on flight data. The intensity of the Earth’s magnetic field determining the direction of the magnetic north is affected and deformed by the current of the motors. We can compensate for this error and calibrate the magnetometer based on knowledge of the position from the flight data. The method can be implemented both online and offline [16]. Another option is to use the position data from several GNSS receivers, which allows for determining the vehicle’s position and rotation [17,18]. GNSS receivers must know the carrier phase. Such an approach has proven to be very effective, but it requires several GNSS receivers that are at least a meter apart.

In our approach, we want to take a closer look at the possibility of calibrating the magnetometer based on the knowledge of the motion vector from GNSS while the vehicle is moving. At each new position, we gain information about the direction vector of the vehicle and its position. Based on this, along with known local declination, we can accurately calculate the direction of the Earth’s magnetic field vector. In our experiments, a car-mounted system is utilized as a test platform. In the case of using a ship or an airplane, we would have to consider the rotation of the vehicle with respect to the direction of the velocity vector due to the influence of sea currents and wind. With ground vehicles like cars, this problem is minimized.

## 2. Magnetometer Calibration Based on GNSS

### 2.1. Magnetometer Error

**Figure 1:**
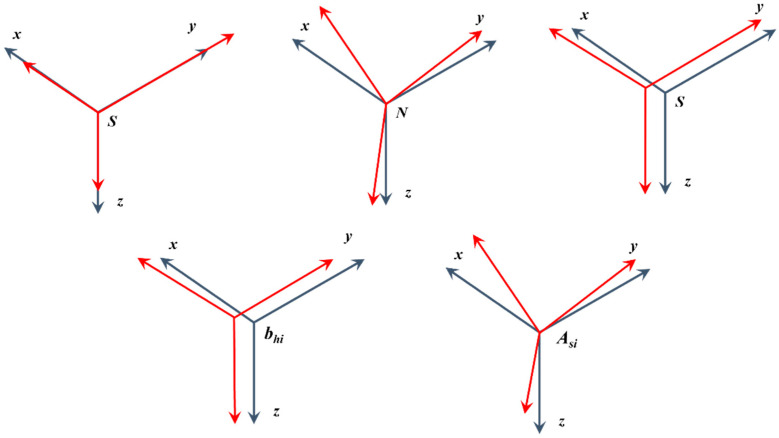
Effect of magnetometer error.

Magnetometers suffer from multiple sources of stochastic and systematic errors. Systematic errors can be divided into two groups. In the first group, there are errors in the sensor itself, such as bias (*b_so_*), nonorthogonality (*N*), and scale factor (*S*). In the second group, there are errors caused by the environment around the sensor, which can be divided into the influence of magnetically hard (*b_h_*_i_) and soft iron (*A_si_*). Figure 1 shows how individual magnetometer errors affect the resulting vector. Soft iron curves the magnetic field lines, thanks to which the sensor measures an ellipsoid instead of a circle. Hard iron is a permanent magnet that creates an ellipsoid (sphere) shift. We can write these errors in Formula (1), which we can rewrite (2). We can combine all errors into the calibration matrix with the size of 3 × 3 and then add the hard iron factor b [2] to the multiplication result. The Formula (1) can be rewritten to (3), where M is a 4 × 3 matrix.

(1) $$h_{m}=SN\left(A_{si}h+b_{hi}\right)+b_{so}$$

(2) $$h_{m}=Ah+b$$

(3) $$h_{m}=Mh$$

The magnetometer will be placed in the vehicle, which is mainly made of ferromagnetic material. Therefore, the calibration deviations will be high. Meanwhile, low-cost magnetometers provide a highly erroneous and noisy output (stochastic error).

### 2.2. GNSS Error

GNSS is a well-known system. We can determine the position of the receiver based on measuring the distance between the receiver and the satellites in the sky while knowing their position. The pseudo-range is based on Formula (4), where *t_r_* − *t_t_* is the total transmission time which we multiply by the speed of light to get our distance from the satellite. Knowing the position of the satellite, we can substitute in Formula (5), where *x_s_*, *y_s_*, *z_s_* are the coordinates of the satellite, *x_r_*, *y_r_*, *z_r_* are our positional coordinates, and *X* is the distance. The GNSS system must be connected to at least four satellites where it calculates four equations with four unknowns—position *x*, *y*, *z*, and time *t* [19].

(4) $$P=\left(t_{r}-t_{t}\right)\times c$$

(5) $$X=\sqrt{{\left(x_{s}-x_{r}\right)}^{2}+{\left(y_{s}-y_{r}\right)}^{2}+{\left(z_{s}-z_{r}\right)}^{2}}$$

The error of GNSS receivers is dependent on several variables. In general, the GNSS error can be removed through Equation (6), where the UERE (User Equivalent Range Error) is defined as the accuracy of pseudo-range measurements, and DOP (Dilution of Precision) describes the geometry of the satellite constellation [20]. In general, we can define that the error can be caused due to: ephemeris error, satellite clock error, ionospheric error, tropospheric error, and multipath and receiver errors. Several algorithms suppress individual GNSS error increments. The parameters with which we describe the accuracy of position estimation are called HDOP (Horizontal DOP) and VDOP (Vertical DOP). In open-sky scenarios, inexpensive GNSS receivers have an accuracy of the order of one meter. In the case of precise localization, we can use a dual-band differential system with support for all currently available GNSS systems (GPS, GLONASS, BeiDou, Galileo).

(6) Error=DOP×UERE

For more accurate positioning, modern approaches are used to guarantee satellite navigation accuracy. A common approach is the use of service areas of satellite-based augmentation systems (SBAS), which use geostationary satellites. Another approach is the Ground-Based Augmentation System (GBAS) which is used in air transport when the aircraft approaches the airport. This service is only local (area near the airport) [21]. The Real-time kinematic positioning (RTK) approach uses multiple receivers with carrier phase determination at multiple frequencies. However, such an approach requires one static station. Another possibility is the PPP approach, which can aim the point with an accuracy below centimeters. It can do this based on the knowledge of satellite orbits and time from GNSS reference stations. However, the initial focus takes up to tens of minutes and requires constant access to GNSS reference stations [22]. We will focus only on cheap sensors that use only SBAS, whose position estimation error in open space is around 2 m.

### 2.3. Calibration Options

**Figure 2:**
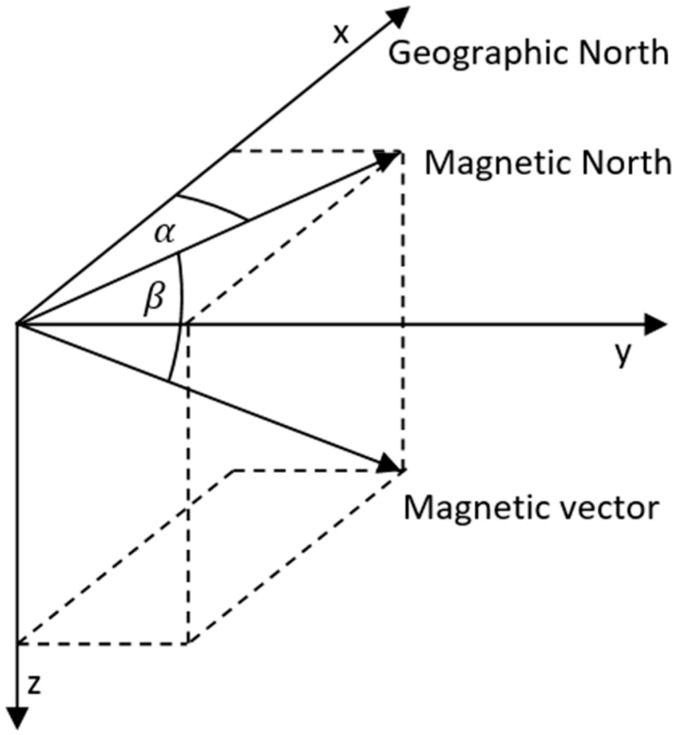
Magnetic field vector.

Magnetometer calibration based on knowledge of the motion vector from GNSS is only possible under certain conditions. As described above, both systems have specific error sources. Suppose we want to calibrate the data based on the knowledge of the path with a certain error from GNSS. In that case, it is necessary to travel the path in the order of hundreds of meters to km so that the error of locating one point is negligible. At the same time, it is necessary to change the path so that it is not always only in one direction. We also need to know the orientation of the sensor for the direction vector. Finally, we need to know the exact values of the magnetic field in the given place and altitude (inclination, declination, magnetic field strength). We can obtain these values based on freely available data or implemented libraries. The direction of the magnetic field vector can be seen in Figure 2.

Based on the knowledge of the path from GNSS, we can calculate the direction vector of movement in the Cartesian coordinate system in Equation (7). The intensity and direction (inclination and declination) of the magnetic field should be known for the given area in which the vehicle is located (vector *M*). Based on this knowledge, one may determine the direction of the earth’s magnetic field vector with respect to the local coordinates of the car. We assume the vehicle moves along a straight path during the calculation. From the motion vector, we calculate the rotation of the global coordinates in Equation (8). Subsequently, we rotate vector B of the known magnetic field of the earth so that we get the exact direction of the magnetic field vector in the local coordinates of the vehicle in Equations (9)–(11).

(7) GPS(lan,lon, alt)→[x,y,z]

(8) $$S_{n}=G_{n}-G_{n-1}\to \psi =atan2\left(y,x\right)$$

(9) $$B=R\cdot M_{n}$$

(10) $$B=\left[\begin{array}{ccc} cos\psi & sin\psi & 0\\ -sin\psi & cos\psi & 0\\ 0 & 0 & 1 \end{array}\right]\;\left[\begin{array}{c} B_{r}\;cos\beta \;cos\alpha \\ B_{r}\;cos\beta \;sin\alpha \\ B_{r}\;sin\beta \end{array}\right]$$

(11) $$B=B_{r}\left[\begin{array}{c} cos\psi \;cos\beta \;cos\alpha +sin\psi \;cos\beta \;sin\alpha \\ -sin\psi \;cos\beta \;cos\alpha +cos\psi \;cos\beta \;sin\alpha \\ sin\beta \end{array}\right]$$

At the same time, we obtain values from the magnetometer *M* (*m_x_*, *m_y_*, *m_z_*). By subtracting the given vectors, we get the sensor error *E* (*e_x_*, *e_y_*, *e_z_*) in Equations (12) and (13). During the calibration phase, we obtain an error vector for each point. After representing the error vectors, we should get a cloud of points that are close to each other. From the data, we need to calculate the gain and bias calibration constants or the calibration matrix. We can obtain the calibration constants of the magnetometer only after passing a certain curved (not straight) path. The error vector is smaller as the speed increases. The algorithm is ineffective when the vehicle is not moving.

(12) E=M−B

(13) $$\left[\begin{array}{c} e_{x}\\ e_{y}\\ e_{z} \end{array}\right]=\left[\begin{array}{c} m_{x}\\ m_{y}\\ m_{z} \end{array}\right]-\left[\begin{array}{c} b_{x}\\ b_{y}\\ b_{z} \end{array}\right]$$

## 3. Experimental Verification

### 3.1. Data Acquisition Module

**Figure 3:**
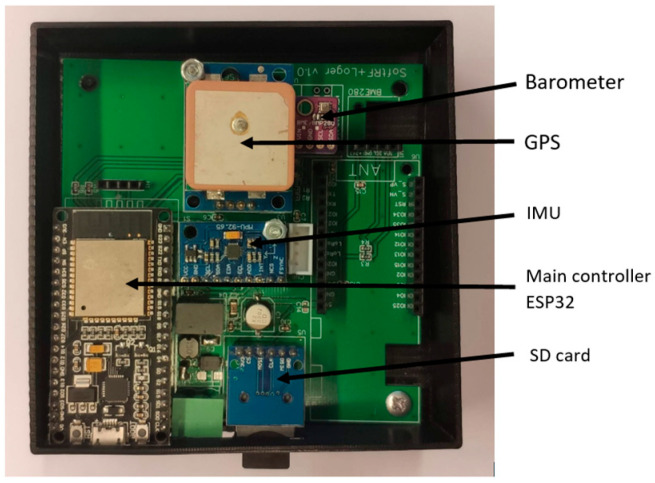
Data acquisition unit.

We needed data for experimental verification. We developed a device for logging data from multiple sensors. The data acquisition unit used the MPU 9250 module for experimental measurements, which combines a three-axis *MEMS* gyroscope, a three-axis MEMS accelerometer, and a three-axis magnetometer. The built-in magnetometer, according to the MPU-9250 datasheet [23]: is a “3-axis silicon monolithic Hall-effect magnetic sensor with magnetic concentrator with an output data resolution of 14 bit (0.6 μT/LSB) and 8 Hz repetition rate”. The GNSS receiver used: Ublox Neo 6P supporting GPS and SBAS: EGNOS (with the capability of other SBAS services which are not suitable for use in the central European area) with velocity accuracy: 0.1 m/s and heading accuracy: 0.5° [24]. The data are stored in the module memory, and after the drive, they are downloaded for processing. The module contains an ESP32 microcontroller, thanks to which it can be connected to a Wi-Fi network, then the module may send the measured data to an FTP server. The module has the option of connecting an OGN (Open Glider Network) transmitter for usage in small aircraft. The data acquisition unit is shown in Figure 3. The module was placed on the dashboard of a passenger car.

### 3.2. Data Processing

We processed the measured data with python scripts using the pandas and NumPy 1.20.2 packages. From the GPS points, we have calculated the direction vectors for each point using Equations (7) and (8) and determined the theoretical axial components of the magnetic vector in the local coordinates of the vehicle in Equation (16). The vectors were calculated only when the vehicle was moving faster than 5 km/h. The faster the vehicle moved, the more accurate estimate of the direction vector was obtained. Calibration is hence not possible when the vehicle is stationary. Based on the obtained error of individual points *E* in Equation (12), we tried three different algorithms for finding calibration constants. The first was the arithmetic average of the error in the given axis, which allows calculating only the bias. Another method was linear regression, based on which we obtained both bias and gain. As the last one, we tried calibration by searching for a calibration matrix that should be able to calibrate the values between the individual axes and Equations (14)–(16).

(14) B=C·V

(15) $$B\cdot V^{T}=C\cdot \left(V\cdot V^{T}\right)$$

(16) $$C=\left(B\cdot V^{T}\right)\cdot {\left(V\cdot V^{T}\right)}^{-1}$$

where *B* is the magnetic field vector from the GPS direction [3 × 1], *V* is the measured vector from the magnetometer [3 × 1], and *C* is the calibration matrix [3 × 4]. The matrix *C* could be of dimension [3 × 3], but the calculation would not consider the bias of the sensor.

**Figure 4:**
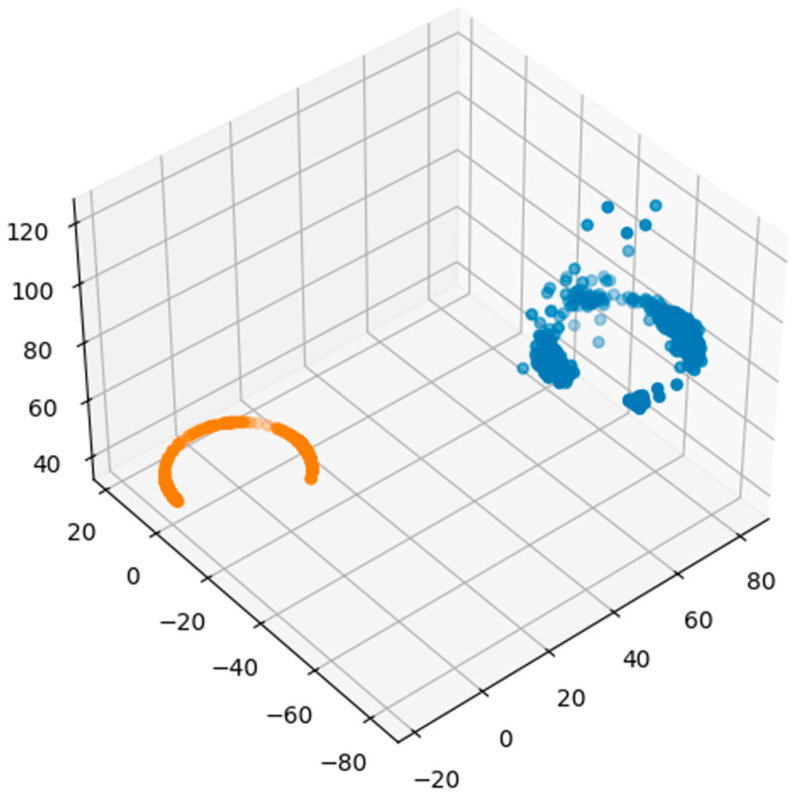
Measured data after a certain path (blue color), calculated data (orange color).

During the data processing, we discovered a strong influence of hard iron in the passenger car cabin. The absolute value of the measured vector was many times greater than the value of the Earth’s magnetic field. This fact can also be seen in Figure 4, where the orange color shows where the points on a certain track should have been, and the blue color shows where they were measured. In the picture, we can also notice certain anomalies that are significantly shifted compared to other measurements. After analysis, we found that these points were created when crossing the iron bridge. In other measurements, they were probably created by the traction lines of railways, trolleybuses, or when passing trucks. As mentioned, the magnetic field is influenced strongly by the environment.

**Figure 5:**
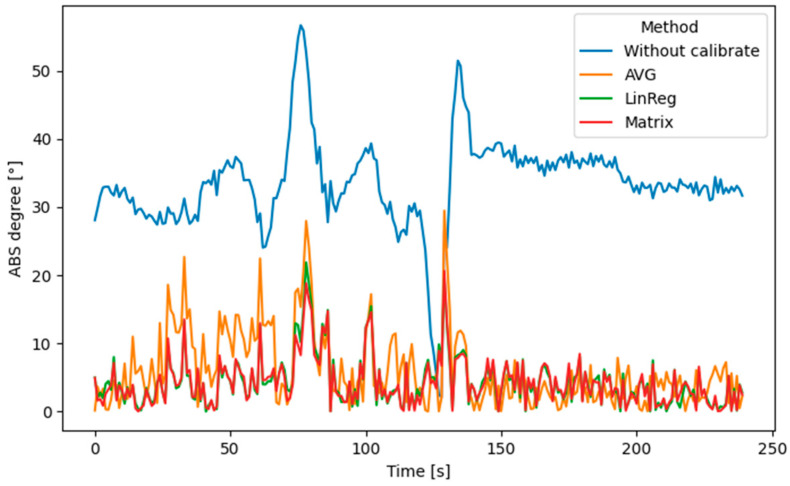
The absolute error of determining magnetic north.

During the calibration phase (1, 2, 3, 4, 5… min), we obtained the error vector *E*, and then, based on the calibration constants, we adjusted the data from the magnetometer for the duration of the journey, which took 4 min. During this evaluation phase, we predicted the position of the vehicle based on the direction from the magnetometer and the speed from the GNSS to show how accurately the data is calibrated. We calculated the difference between the predicted direction vector (azimuth) and the real one from the GNSS during the prediction period. The progress of the error during the prediction can be seen in Figure 5. The error does not contain drift. The total error was reflected in the RMSE values, which for this case, were as follows

- without calibration: 33.6°,
- averaging error (AVG): 6.0°,
- linear regression (LinReg): 4.39°
- and calibration matrix estimation (Matrix): 4.33°.

**Figure 6:**
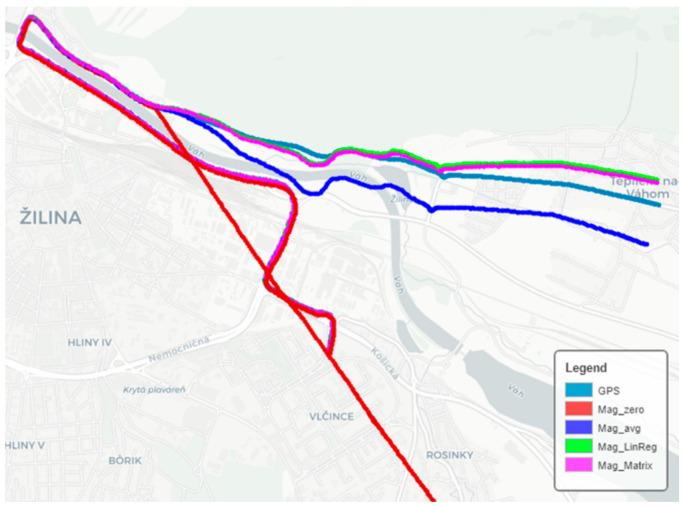
Map calibration and subsequent position prediction.

The prediction of the position, with different types of search for calibration constants, can be seen in Figure 6, where the magnetometer was calibrated for 4 min, and then the position of the vehicle was predicted for 4 min. The direction of the uncalibrated data (red color) was almost always in the same direction. This phenomenon was caused by the car’s hard ferromagnetic material. The absolute value of the measured vector was around 120 μT, while the intensity of the magnetic field in the measurement locations was 49.5 μT. At the end of each measurement, we placed a compass at the location of the sensor and recorded its value. This coincided with the information from the uncalibrated magnetometer. The magnetic field in the car is different in every place. When measuring with a compass in several places in the vehicle, the needle is always pointed in a different direction.

## 4. Achieved Results

**Figure 7:**
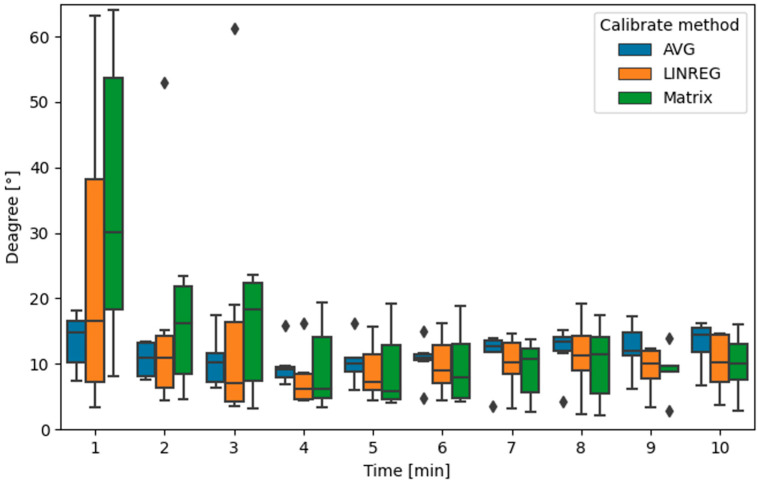
Boxplot of calibration results.

In total, we performed 20 measurements and calibrated the data at 1–10 min intervals. Then, we determined the direction vector of the calibrated magnetometer. The RMSE error for individual calibration methods was determined and statistically processed. The results of the measurements were comparable to each other. In the case of shorter calibration (1–3 min), the most successful calibration was through the arithmetic mean or linear regression. For a longer time (more than 3 min), the algorithm was more accurate through the search of the calibration matrix. The results can be seen in Figure 7. The boxplot was created using the seaborn 0.11.2 library. The average error in determining the direction based on the magnetometer was around 10° after a calibration time of at least 5 min. The result was strongly dependent on the track during calibration. The calibration result was more accurate if the vehicle traveled in several directions during the calibration. The path during the calibration in Figure 6 was zigzag, and the result of the calibration after 5 min was 4.33° when using the search of the calibration data through the matrix.

## 5. Conclusions

The calibration of the magnetometer is very important as the magnetic field is strongly influenced by the environment. The calibration method proposed by us is especially advantageous for using the magnetometer in inertial navigation in combination with GNSS. The magnetometer or other sensors can be calibrated based on the measurement of the direction vector from GNSS if the vehicle is moving. In case of signal failure, we can predict the position based on the INS, whose sensors can be cheap. Unlike a gyroscope, a magnetometer is not subject to drift. In our tests, we achieved an average deviation in magnetometer accuracy of 10° with a calibration time of at least 5 min, using linear regression and matrix calibration. This error may seem large, but you have to remember that in a passenger car, the magnetic field is strongly influenced by the surroundings. The magnetometer had deviations when passing traction lines and iron bridges. It also had minor deviations when driving around trucks. The magnetic field in the car cabin was many times greater than the real value of the Earth’s magnetic field at our latitude and altitude. Such calibration is strongly dependent on the traveled path; in case the data was calibrated only during a section on a straight path, the calibration results were imprecise but still better than in the case of data without calibration. Such a calibration approach can be used in all mobile objects with GNSS or other localization. However, it is necessary to know the current rotation of the sensor relative to the track. In the further direction of the project, we want to look at the possibilities of gyroscope and magnetometer calibration using the direction vector from GNSS. By fusing the data from the gyroscope and the magnetometer, we would be able to determine the direction of the vehicle more accurately. Using the estimated direction vector and speed of the vehicle, we can predict the position. The speed data can be obtained from the vehicle’s control unit, as cheap accelerometers are too inaccurate for integrating the speed of an object, and there is a thermal drift that we cannot address.

## Data Availability

Data is available upon request from the corresponding author.
